# Hematopoietic Overexpression of FOG1 Does Not Affect B-Cells but Reduces the Number of Circulating Eosinophils

**DOI:** 10.1371/journal.pone.0092836

**Published:** 2014-04-18

**Authors:** Camille Du Roure, Aude Versavel, Thierry Doll, Chun Cao, Vincent Pillonel, Gabriele Matthias, Markus Kaller, Jean-François Spetz, Patrick Kopp, Hubertus Kohler, Matthias Müller, Patrick Matthias

**Affiliations:** 1 Friedrich Miescher Institute for Biomedical Research, Basel, Switzerland; 2 Novartis Institutes for Biomedical Research, Basel, Switzerland; 3 Faculty of Sciences, University of Basel, Basel, Switzerland; Muséum National d'Histoire Naturelle, France

## Abstract

We have identified expression of the gene encoding the transcriptional coactivator FOG-1 (Friend of GATA-1; *Zfpm1*, *Zinc finger protein multitype 1*) in B lymphocytes. We found that FOG-1 expression is directly or indirectly dependent on the B cell-specific coactivator OBF-1 and that it is modulated during B cell development: expression is observed in early but not in late stages of B cell development. To directly test *in vivo* the role of FOG-1 in B lymphocytes, we developed a novel embryonic stem cell recombination system. For this, we combined homologous recombination with the FLP recombinase activity to rapidly generate embryonic stem cell lines carrying a Cre-inducible transgene at the *Rosa26* locus. Using this system, we successfully generated transgenic mice where FOG-1 is conditionally overexpressed in mature B-cells or in the entire hematopoietic system. While overexpression of FOG-1 in B cells did not significantly affect B cell development or function, we found that enforced expression of FOG-1 throughout all hematopoietic lineages led to a reduction in the number of circulating eosinophils, confirming and extending to mammals the known function of FOG-1 in this lineage.

## Introduction

The development of specialized hematopoietic cells from self-renewing hematopoietic stem cells proceeds through a number of precursor stages with progressively restricted differentiation potential and requires a complex interplay of transcription factors and epigenetic modifiers. These regulators are responsible for orchestrating the establishment of lineage-specific gene expression patterns that underlie cellular differentiation (reviewed in [1], [2]). While many factors involved in this process are already known, a complete molecular understanding is still missing. Friend of GATA-1 (FOG-1), which is encoded by the *Zfpm1* (*Zinc finger protein multitype 1*) gene, was previously thought to be expressed primarily in cells of the erythroid and megakaryocytic lineages, where it is essential for differentiation [3], [4]. FOG-1 is a zinc finger protein initially identified as an interacting partner of GATA factors which contributes to activation or repression of their target genes [3], [5], [6]. FOG-1 also interacts with the C-terminal binding protein (CtBP), mainly described as a corepressor and the nucleosome remodelling and histone deacetylase repressive (NuRD) complex and thus makes a link between transcription factors and chromatin modifiers. FOG-1 also activates or represses gene transcription by facilitating binding of GATA factors to DNA [7], recruiting chromatin remodelling complexes [5], [8], or by stabilizing tissue-specific chromatin loops [9]. FOG-1 is expressed at high level in multipotent progenitors, erythroid and megakaryocytic cells, low level in lymphoid and haematopoietic stem cells; it is absent in myeloid lineages [3]. *Zfpm1*-deficient mice lack megakaryocytes and show severe defects in erythropoiesis, leading to embryonic lethality [4]. FOG-1 also plays a role in the T-lineage by repressing GATA-3-dependent induction of Th2 development [10], [11]. Interestingly, overexpression of FOG-1 in avian eosinophils, which do not normally express FOG-1, reprograms these differentiated cells into multipotent cells [12], reminiscent of the reprogramming of B-cells into macrophages following ectopic expression of C/EBPalpha and C/EBPbeta [13], [14]. Thus, FOG-1 is essential for specific branches of the haematopoietic system, and its inappropriate expression leads to abnormal cell differentiation.

Strikingly, we have identified relatively high FOG-1 expression in early B-lymphocytes, and low or lack of expression in late developmental stages such as mature B-cells and plasma cells. In analogy to some of the systems mentioned above, we were intrigued by the regulated expression of FOG-1 during B cell development and hypothesized that the downregulation of FOG-1 might be a necessary step for proper differentiation and function of mature B cells. We therefore set out to test this hypothesis and for this made use of a novel transgenic mouse model strategy based on recombination mediated cassette exchange (RMCE, [15], [16]) which we had designed to generate mice with conditional overexpression of any cDNA. Using this system, we generated transgenic mice in which FOG-1 expression was enforced at a physiologically relevant level in mature B-cells or in the entire hematopoietic system. We found that sustained FOG-1 expression in mature and late B cells did not affect their development or function, contrary to our hypothesis. In contrast, overexpressing FOG-1 in the whole hematopoietic system led to a reduction in the number of circulating eosinophils, confirming and extending to mammals the previously reported role of FOG-1 in repressing avian eosinophil development.

## Materials and Methods

### Ethics statement

Animal experimentation was carried out according to regulations effective in the canton of Basel-Stadt, Switzerland. All experimental procedures were approved by the Animal Committee of the Friedrich Miescher Institute for Biomedical Research and the Veterinary office of the Kanton Basel Stadt.

### Generation of the targeting pR26-SA-FRT-Hygro^r^ vector

The backbone of a pre-existing targeting vector pR26-STOP-FRT-Hygro [17] was adapted to allow transcription from the endogenous *Rosa26* promoter. After removal of the unwanted sequences, the backbone vector contained two homology arms and the hygromycin B resistance gene flanked by an FRT3 and an FRTwt sites in 5′ and 3′, respectively. The 5′ and 3′ homology arms correspond to Chr6: 113 024 284–113 026 000 and to Chr6: 113 021 493–113 024 090 using the mm9 assembly on the UCSC genome browser. The integration site maps about 2 kb downstream of the insertion point obtained with the targeting vector pROSA26-1 [18]. The splice acceptor sequence (SA) of the STOP-eGFP-Rosa26TV vector (Adgene plasmid 11739) was PCR amplified and cloned upstream of the FRT3 site [19]. The STOP sequence from Adgene plasmid 11739 is based on SV40 polyA sites.

### Generation of the control and FOG-1 donor vectors

The backbone of the donor vector, containing the FRT3 site, a polyA sequence and the FRTwt site, was derived from the FRT3-CAG-lox-stop-lox-Enpp1-tkNeo-FRTwt [17] vector and was further modified as follows. The loxP-Neo-STOP-loxP cassette was PCR amplified from the STOP-eGFP-Rosa26TV vector (Adgene plasmid 11739) with 5′-tagccctaggcttcgcggtctttccagtggt-3′ and 3′-atgcaccggtcttcggtaccgaattgatcg-5′ containing an AvrII and an AgeI site, respectively. This fragment was cloned downstream of the FRT3 site using SpeI and AgeI sites. The IRES-hCD2t fragment was obtained from the pBS-IRES-hCD2t vector (kindly provided by M. Busslinger, Vienna, [20]) and cloned downstream of the loxP-Neo-STOP-loxP cassette. The resulting control donor vector FRT3-loxP-Neo-STOP-loxP-IRES-hCD2t-FRTwt harbors a unique NotI site in between the second loxP site and the IRES sequence. The FlagFOG-1 cDNA which is encoded by the *Zfpm1* gene was obtained from a pcDNA3-FlagFOG-1 vector (kindly provided by M. Crossley, Sydney) and cloned in the NotI site of the control donor vector, resulting in the 10.9 kb FOG-1 donor vector: FRT3-loxP-Neo-STOP-loxP-FlagFOG-1-IRES-hCD2t-FRTwt.

### Targeting of ES cells

The SacI-linearized targeting pR26-SA-FRT-Hygro^r^ vector was electroporated into 129 sv jae ES cells. Electroporated ES cells were then selected with 0.1 mg/ml hygromycin B. 480 hygromycin-resistant clones were collected, and five potentially successfully recombined clones were identified by PCR screening using the following primer pair (0F: ctactggaaagaccgcgaag, 0R: tacctttctgggagttctctgc, 2 kb product).

### Southern blot analysis

Verification of ES cell targeting: 10 µg of genomic DNA was analyzed by standard Southern blotting. Genomic DNA was restricted with BamHI, PstI or PvuII to confirm the 5′, 3′ and single integration of the targeting vector, respectively. The 5′ and 3′ probes were PCR amplified from genomic DNA with the following primers: 5′ fwd: cgcctaaagaagaggctgtg and 5′ rev: gactggagttgcagatcacg; 3′ fwd: agccatctgggccttttaac and 3′ rev: aagggcacagacaatccttc. The 5′ probe highlighted a 5.8 kb wild-type and a 4.9 kb targeted bands. The 3′ probe detected a 6.5 kb wild-type and a 7.5 kb targeted bands. The internal hygromycin probe was obtained from the targeting vector and highlighted an 8 kb band.

Verification of the RMCE targeting: 20 µg of genomic DNA was analyzed by standard Southern blotting. Genomic DNA was digested with PvuII or BglI to confirm the 5′, internal and 3′ single integration of the donor vector, respectively. The 5′ and 3′ probes were PCR amplified from genomic DNA with the following primers: 5′ fwd: cgcctaaagaagaggctgtg and 5′ rev: gactggagttgcagatcacg and 3′ fwd: ggacaggacagtgcttgtttaagg and 3′ rev: acaccacaaatgaacagtgccaag. The 5′ probe highlighted a 5.8 kb wild-type and a 6.3 kb targeted bands. The 3′ probe detected a 6.5 kb wild-type and a 6.2 kb targeted bands. The internal neomycin probe was obtained by PCR from the control donor vector using the following primers: Neo fwd: gaactcgtcaagaaggcgatagaag and Neo rev: gaacaagatggattgcacgcagg. It highlighted a 3.2 kb and a 2.4 kb bands in the control clone and FOG-1 clone, respectively in addition to a 6.3 kb targeted band detected in both clones.

### Recombinase-mediated cassette exchange: FLP-mediated recombination of the control and FOG-1 donor vectors into the pre-targeted ES cells

The pre-targeted R26^Hygro^ ES cells were thawed and cultured for 2 days on feeders in ES cell culture medium without hygromycin. 0.8×10^6^ cells in a 6 cm dish were then transfected with a FLP-expressing vector together with the FOG-1 donor vector (FRT3-loxP-Neo-STOP-loxP-FlagFOG-1-IRES-hCD2t-FRTwt), or the control donor vector (FRT3-loxP-Neo-STOP-loxP-IRES-hCD2t-FRTwt) using Effectene reagent according to the manufacturer's instructions. One day later, the medium was replaced by ES cell medium containing 0.2 mg/ml geneticin. Ten days later, 48 or 94 colonies were picked and single-cell suspensions made by trypsin treatment. Each colony was then plated in medium containing geneticin only or geneticin and hygromycin (0.1 mg/ml). Five days later, colonies that were geneticin resistant and hygromycin sensitive were picked and seeded in 35 mm dishes for further expansion.

Clones of interest were checked by PCR for correct insertion (see Figure S2A for a scheme of the strategy). The 5′ insertion was verified by primer pair 1 (1F: 5′-aactcttcgcggtctttcc-3′ and 1R: 3′-tctggattcatcgactgtgg-5′) and the 3′ insertion by primer pair 2 (2F: 5′-gccttcttgacgagttcttctgag-3′ and 2R: 3′-gaaggacggtacaccagagaac-5′). A primer pair 3 (3F: 5′-aactcttcgcggtctttcc-3′ and 3R: 3′-gactttccacacctggttgc-5′) and a primer pair 4 (4F: 5′-agagcttggcgtaatcatgg-3′ and 4R: 3′-cgtaagggattactcggtga-5′) amplifying only products in the unrecombined allele were used as negative controls. For checking the integration of the neomycin cassette by PCR, the same primers than those used for the synthesis of the internal neomycin probe and specified above were used. The integration of the hCD2t sequence was checked by PCR with the following primers: fwd: tctgaagaccgatgatcagg and rev: tcattacctcacaggtcagg. The primer pair 2′ (2F′: 5′-aacagatggtccccagatgc-3′ and 2R′: 3′-agtggctcattagggaatgc-5′) was used for genotyping the R26^FOG-1^ mice.

### Cre-expression in the recombined ES cells

10^6^ R26^FOG-1^ ES cells were electroporated with a Cre-expressing vector (pCAGS-nlsCre-PGK-Puro, kindly provided by D. Schübeler, Basel). Neomycin-selection was performed at a concentration of 0.2 mg/ml geneticin for 48 hrs in absence of feeders.

### Mouse work

The mice were housed in groups of one to six animals at 25°C with a 12-h light-dark cycle (12 h light, 12 h dark) and were fed a standard laboratory diet containing 0.8% phosphorus and 1.1% calcium (NAFAG 890; Kliba, Basel, Switzerland). Food and water were provided *ad libitum*.

R26^FOG-1^ ES cells were used to generate chimeric mice which were then crossed with C57BL/6J mice to generate transgenic animals. The R26^FOG-1^ mice were subsequently crossed with different Cre-expressing mouse lines to obtain overexpression of FOG-1 in specific B cell subpopulations or throughout hematopoietic lineages. In particular, they were crossed with Cd23-Cre [20], Vav-iCre [21] or mb1-Cre mice [22] to obtain R26^FOG-1^:Cd23-Cre, R26^FOG-1^:Vav-iCre or R26^FOG-1^:mb1-Cre mice, respectively.

Mice or targeted ES cells will be made available upon request.

### Cell culture and RT-PCR analysis

Cells were cultured in a humidified tissue culture incubator set up at 5% CO_2_ and 37°C. Abelson lines (OBF-1 wt or OBF-1^−/−^), B3, A20J, X63Ag8 and J558L cell lines were cultured in RPMI 1640 completed with 10% heat-inactivated FCS, 1% penicillin-streptomycin and 4 mM L-glutamine. Total cellular RNA was extracted using RNeasy Mini Kit (Qiagen), DNaseI treated and reverse transcribed using oligo(dT) and SuperScript II RT (Invitrogen) kit according to standard procedures. Subsequent quantitative real-time PCRs were performed with the MESA GREEN qPCR MasterMix Plus for SYBR (Eurogentec) on an ABI prism 7000 instrument. The FOG-1 primers were 5′-ccaactgtgaacgccatctc-3′ and 3′-gatctcacccttggagcctg-5′. The primers specific for transgene-derived FOG-1 (FlagFOG-1) were 5′-atggactacaaggacgacg-3′ and 5′-tccatggccttggcttcttc-3′. The RNA polymerase II (RPII) primers were 5′-aggagcgccaaatgccgataa-3′ and 5′-aggagcgccaaatgccgataa-3′. The GAPDH primers were 5′-TGCACCACCAACTGCTTAG-3′ and 5′-TGGAAGAGTGGGAGTTGCTG-3′. The moue beta actin primers were: 5′-ctaaggccaaccgtgaaag-3′ and 5′-accagaggcatacagggaca-3′.

### Western blot analysis

Proteins were separated on a 7–8% SDS–PAGE, transferred to a PROTRAN Nitrocellulose Transfer Membrane (Whatman) or to an Immobilon-P Membrane PVDF (Millipore) and immunoblotted with the appropriate primary and secondary antibodies. The following antibodies were used: anti-FOG-1 (Santa Cruz, sc-9362), anti-β-tubulin (Sigma, T4026), anti-actin (NeoMarkers, MS-1295-P1), anti-goat IgG, HRP (Abcam, ab7125), anti-mouse IgG, HRP (GE Healthcare, NA931V), anti-goat 680 and anti-mouse 680 (Molecular Probes). Signals were detected either with Amersham HyperFilm ECL (GE Healthcare) or quantified using a LI-COR Odissey instrument and the Odissey 2.1 software (Biosciences).

### Flow cytometry analysis

Cells were stained in PBS-3%FCS for 30 min on ice with the following antibodies: anti-B220 (BD 553092), anti-CD25 (BD 553050), anti-IgM (Southern biotechnology 1140-02), anti-TER119 (BD 557915), anti-CD71 (BD 553267), anti-CD4 (BD 553729), anti-CD8 (BD 553032), and anti-hCD2t (R&D FAB 18561P). For cell sorting, the following antibody combinations were used: ProB cells, B220+, cKit+, CD25−, IgM−; large or small Pre B, B220+, cKit−, CD25+, IgM−; immature B, B220+, IgM^low^; splenic mature B, B220+, IgM^high^. Cells were analyzed on a Becton Dickinson FACSCalibur or sorted on a Cytomation MoFlo instrument

### Mature B-cell activation

10^6^ splenic mature B-cells purified using CD43-magnetic beads (Miltenyi Biotec) were cultured for 4 days in the presence of IL4 (10 ng/ml) and/or LPS (5 µg/ml) and/or anti-CD40 antibody (1 µg/ml; BD 553721).

### ELISA assay

IgM or IgG1 antibody titers in the mature B-cell activation cultures were determined by standard ELISA protocol.

### Full blood analysis

Tail blood samples were collected in EDTA-coated tubes and analyzed with a Sysmex XT-2000iV blood analyzer.

### Statistical analysis

Where appropriate, standard error of the mean is presented. Statistical significance (p value<0.05) was determined by performing t-test analysis.

## Results

### Identification of FOG-1 expression in B cells

**Figure 1:**
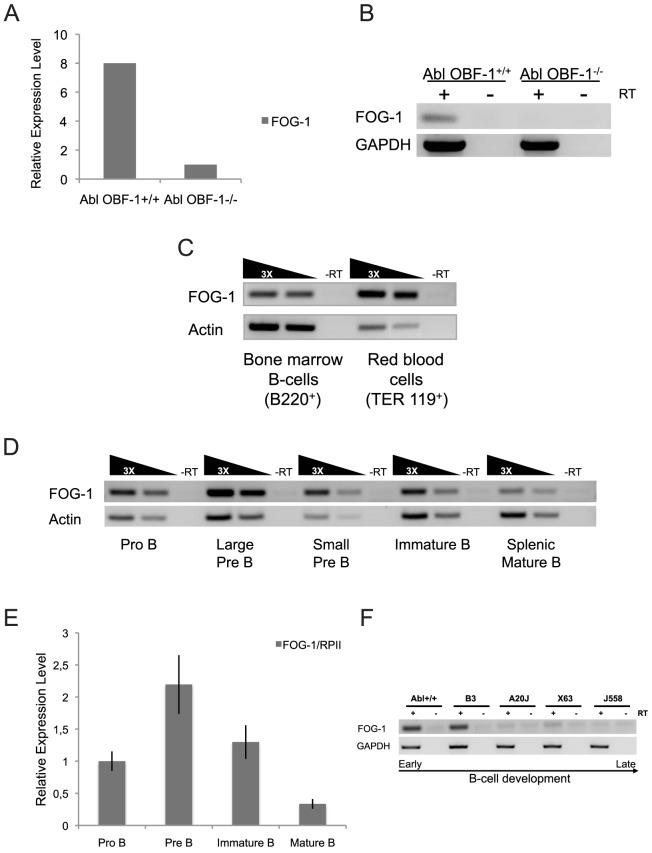
FOG-1 is expressed in a regulated manner during B-cell development. (A) Gene expression level of FOG-1 determined by Affymetrix microarray in wild-type or *OBF-1^−/−^* Abl Pro-B-cells [25]. (B) Reverse transcriptase (RT)-PCR analysis of FOG-1 mRNA level in wild-type or *OBF-1^−/−^* Abl Pro-B-cells. GAPDH mRNA amount was used as a control. +, − refer to cDNA synthesis reactions performed in the presence or absence of reverse transcriptase (RT), respectively. (C) mRNA expression level of FOG-1 in bone marrow B-cells (B220^+^) and in red blood cells (TER 119^+^) determined by semi-quantitative RT-PCR. Actin mRNA amount was used as a reference. (D) mRNA expression level of FOG-1 during B-cell development determined by semi-quantitative RT-PCR. The analysis was performed on primary Pro-B, large Pre-B, small Pre-B, immature-B and splenic mature-B cells. Actin mRNA amount was used as a reference. (E) mRNA expression level of FOG-1 measured by real-time RT-PCR during the B-cell development. The analysis was performed on primary B-cells as in (D). RNA Polymerase II mRNA was used as a reference. (F) mRNA expression level of FOG-1 in B-cell lines representing different stages of B-cell development, determined by RT-PCR. From early to late stages, the analysis was performed on the five following cell lines: wild-type Abelson (Abl+/+), B3, A20J, X63 and J558. GAPDH mRNA was used as a control.

In previous studies from our laboratory microarray analysis had been used to determine expression profiles of Pre-B-cells lacking the transcriptional coactivator OBF-1 [23], [24] and transgenic thymocytes overexpressing OBF-1; these experiments identified several genes that were downregulated in null cells [25] or upregulated in overexpressing cells [25], [26]. Among these genes, we identified *Zfpm1*, which encodes the developmental regulator FOG-1 (Fig. 1A, 1B and data not shown). *Zfpm1* had also been identified as a gene consistently activated or repressed in *Ebf1* (early B-cell factor-1) gain- and loss-of-function experiments, respectively and ChIP-Seq data demonstrated that Ebf1 binds to the promoter of *Zfpm1* within 10 kb of the transcription start site [27].

In agreement with previous results [3], we detected a lower expression level of FOG-1 in total bone marrow B-cells than in red blood cells (Fig. 1C). Microarray and quantitative RT-PCR analysis demonstrated that FOG-1 was expressed from Pro-B-cell to immature B-cell stages at a relatively high level and was downregulated in mature B-cells and plasma cells. This specific expression pattern was observed in primary cells and also in cultured cell lines representative of different B-cell developmental stages (Fig. 1D, 1E and 1F).

Together these results show that FOG-1 is expressed in a regulated manner during B cell development and suggest that this factor may play a role not previously appreciated in this lineage. To examine this in greater detail, we wished to test the effect of overexpressing FOG-1 in B cells, in particular in late stages, hypothesizing that this might affect their differentiation or function. For this, we made use of a system that we had designed to generate mice overexpressing a gene of interest in a conditional manner.

### Pre-targeting of the *Rosa26* locus

**Figure 2:**
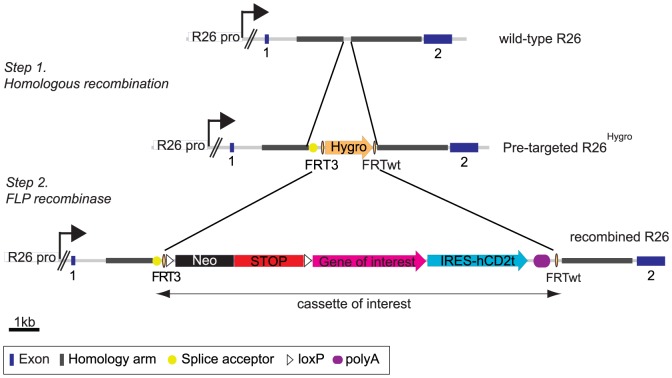
Strategy for the rapid generation of conditionally overexpressing ES cells. Schematic representation of the wild-type *Rosa26* (R26) locus, the pre-targeted R26^Hygro^ and the recombined R26 alleles. The *Rosa26* locus was first pre-targeted by homologous recombination with a cassette containing a splice acceptor and a hygromycin B resistance gene flanked by a FRT3 site in 5′ and a FRTwt site in 3′ to generate the pre-targeted R26^Hygro^ allele (step 1). The hygromycin B cassette is then replaced by the cassette of interest via the FRT sites using transient expression of the FLP recombinase (step 2). Our cassette of interest contains a loxP-Neo-STOP-loxP cassette for neomycin selection of ES cells and conditional expression of the transgene, and an IRES-hCD2t sequence to monitor expressing cells. Notably, Cre-mediated recombination will excise the neomycin gene from the targeted allele in the transgenic mice. Neo: neomycin resistance gene. R26 pro: promoter of the R26 gene.

Our strategy has been to generate ES cells pre-targeted at the *Rosa26* (R26) locus, so that appropriate expression constructs can rapidly be inserted by recombination mediated cassette exchange (RMCE, see Figure 2). We chose to use the *Rosa26* locus, which was first described in a gene trap experiment and was shown to be expressed in the whole mouse [28]. This locus is believed to encode two non-coding transcripts and an antisense transcript of unknown function and can be used to drive the expression of any cDNA [28]. In the original gene trap experiment, the insertion of a splice acceptor and a promoter-less cDNA in intron 1 of the gene led to expression of this cDNA from the endogenous *Rosa26* promoter. For these reasons, we decided to target the *Rosa26* locus and we used 5′ and 3′ homology arms located in intron 1. The targeting vector containing a splice acceptor sequence to allow expression from the endogenous *Rosa26* promoter and an hygromycin B resistance gene flanked by FRT3 and FRTwt sites was linearized and introduced by electroporation into 129svjae ES cells to generate the pre-targeted R26^Hygro^ allele (Figure 2, step 1 and Figure S1A). 480 hygromycin-resistant ES cell clones were first screened by PCR using a forward primer located upstream of the 5′ homology arm and a reverse primer located in the hygromycin cassette. Four clones (1–4, Figure S1B) showed the expected 2 kb band for successful homologous recombination, as compared to the aberrant product obtained for clone 5. To further confirm the correct homologous recombination, we performed Southern blot analysis using 5′ and 3′ probes with BamHI or PstI-digested genomic DNA blots. Clones 1–4 showed the expected 4.9 kb band for correct integration of the selection cassette in 5′ (Figure S1C), as well as the 7.5 kb band for correct integration at the 3′ end (Figure S1D). As a control, clone 5 only showed the wild-type bands. To ensure that a single copy was integrated in the genome, an additional Southern blot analysis was performed with a hygromycin probe and PvuII-digested genomic DNA blots. Clones 2–4 showed a single band at the expected size, whilst clone 1 showed an additional smaller band, suggesting multiple insertions of the transgene (Figure S1E). Thus, our targeting vector was successfully homologously recombined into the *Rosa26* locus to generate the pre-targeted R26^Hygro^ allele. Clone 4 was selected as our R26^Hygro^ ES cell clone for further use.

### Efficient recombination of the FOG-1 cassette in the pre-targeted R26^Hygro^ locus

**Figure 3:**
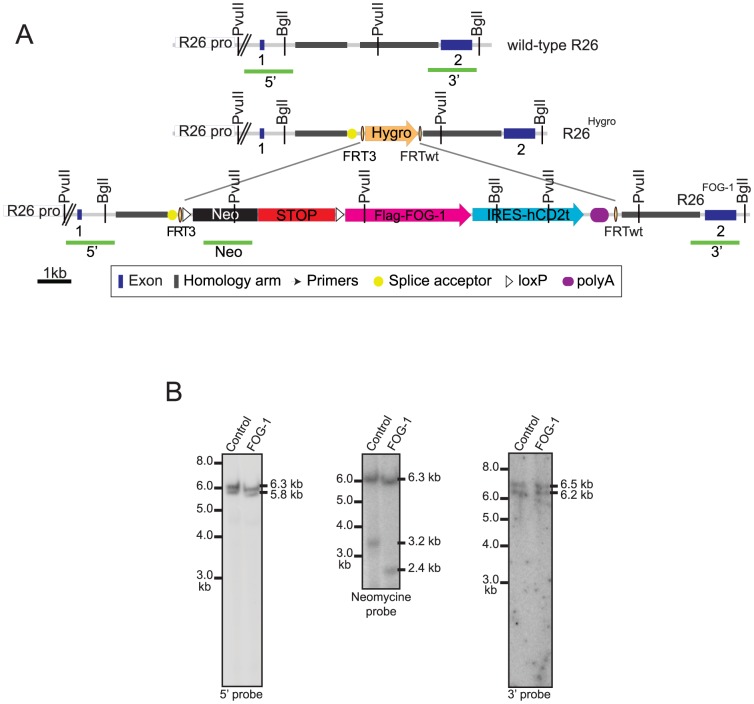
Correct recombination at the pre-targeted R26^Hygro^ allele by RMCE. **A**. Schematic representation of the wild-type R26, pre-targeted R26^Hygro^ and recombined R26^FOG-1^ alleles. In the FOG-1 cassette, the neomycin resistance gene (Neo, black) and the STOP sequence (red) are flanked by LoxP sites (white triangles) to allow conditional expression. The cDNA encoding FlagFOG-1 (pink) followed by an IRES sequence and the sequence coding for hCD2t (pale blue) were inserted downstream. A polyA signal was placed at the 3′ end of the hCD2t coding sequence (blue oval). The probes (green bars) used for Southern blot analysis are shown. **B**. Correct insertion of the vectors was confirmed by Southern blotting. To test the 5′ boundary, PvuII-digested ES cell genomic DNA was hybridized with the radioactively labeled 5′ probe to detect the wild-type (5.8 kb) and the targeted (6.3 kb) bands (Left panel). To verify single-copy insertion, PvuII-digested DNA was hybridized with a radioactively labeled internal probe to detect the 3.2 kb or 2.4 kb targeted bands in control or FOG-1 clones, respectively, in addition to the 6.3 kb targeted band (Middle panel). To test the 3′ boundary, BglI-digested DNA was hybridized with the radioactively labeled 3′ probe to detect the 6.5 kb wild-type and the 6.2 kb targeted bands (Right panel).

The combined use of the heterospecific FRT3/FRTwt sites allows replacement of a target DNA by an incoming plasmid donor cassette upon transient FLP expression [29]. Hence, any cassette of interest flanked by FRT3/FRTwt sites can readily be introduced into pre-targeted R26^Hygro^ ES cells upon transient expression of the FLP recombinase (Figure 2, step 2). Inducible expression cassettes contain a loxP-Neo-STOP-loxP sequence upstream of the cDNA of interest to allow neomycin selection of the recombined clones and Cre-dependent expression of the transgene from the endogenous *Rosa26* promoter. The cassettes also contain an internal ribosome entry site (IRES) sequence derived from the Encephalomyocarditis virus placed downstream of the cDNA of interest to allow the concomitant expression of a “reporter” gene (here a truncated version of the human cell surface marker CD2, hCD2t), and selective monitoring of the recombined cells. In order to study the effect of enforced expression of FOG-1 in transgenic mice, two donor vectors were generated: (i) a control donor vector with loxP-Neo-STOP-loxP and the IRES-hCD2t sequences, but no cDNA (Figure S2A), (ii) the FOG-1 donor vector which contained a cDNA encoding Flag-tagged FOG-1 downstream of the loxP-Neo-STOP-loxP sequence and upstream of the IRES-hCD2t sequence (Figure 3A and Figure S2A).

To test the efficiency of our system, RMCE experiments were performed in R26^Hygro^ ES cells: cells were transfected with either the FOG-1 donor vector or the control donor vector, together with an expression vector encoding the FLP recombinase. The R26^Hygro^ ES cells are resistant to hygromycin B and, upon successful RMCE, become sensitive to this antibiotic, while acquiring neomycin-resistance (see Figure 2). In two independent RMCE experiments, a total of 142 Neo^R^ colonies were picked for each vector and were tested for hygromycin B resistance, as described in the Materials and Methods section. We found that at this step 48.5% of the FOG-1 colonies and 62.5% of the control colonies were both neomycin-resistant and hygromycin-sensitive, indicative of successful RMCE. We next selected 12 Neo^R^/Hygro^S^ clones of each kind (control or FOG-1 vector) for an extensive PCR analysis which demonstrated that all the clones analyzed were properly recombined (Figure S2A–B). Successful integration of the neomycine cassette and of the hCD2t cassette in the genome of these ES cell clones was also tested and all clones were positive for these PCRs as well (Figure S2C–D). Thus, using the system described here, we efficiently recombined a large cassette of 5.0 kb or 8.0 kb at the R26^Hygro^ allele of the pre-targeted ES cells to generate ES cell clones carrying the R26^Control^ allele or R26^FOG-1^ allele, respectively. Based on the extensive PCR analysis presented here, 100% of the Neo^R^/Hygro^S^ clones appear to be correctly recombined upon RMCE. We next chose one control clone and one FOG-1 clone for verifying the successful RMCE by Southern blot analysis and used genomic digests and probes allowing us to interrogate the 5′ and the 3′ boundaries, as well as the copy number. This analysis also confirmed the correct recombination of the targeting vectors into the *Rosa26* locus (Figure 3A–B).

**Figure 4:**
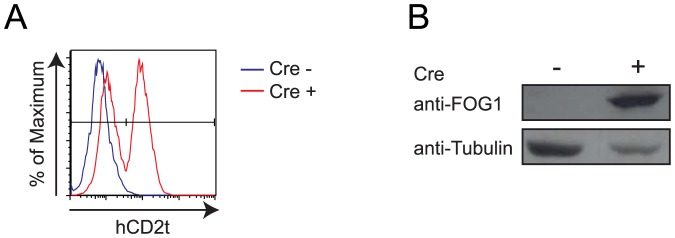
Cre-dependent expression of hCD2t and FOG-1 in recombined ES cells. R26^FOG-1^ ES cells were electroporated with a Cre expressing vector. Cre-induced expression of hCD2t and FOG-1 was detected by flow cytometry (**A**) and western blot analysis (**B**), respectively.

Before generating mice, we tested the Cre-inducible expression of FOG-1 and hCD2t in our recombined FOG-1 clone. To this end, a Cre-expressing vector was transiently transfected in the targeted ES cells and expression of hCD2t and FOG-1 was tested two days later by flow cytometry and western blot analysis, respectively. As expected, the recombined R26^FOG-1^ ES cells expressed hCD2t and FOG-1 only when Cre was expressed (Figure 4A and 4B). The partial expression of hCD2t observed by flow cytometry is due to the experimental settings: here, transfected ES cells were only selected for a short period of time to avoid FOG-1-induced cell cycle arrest in ES cells [30]. As a result of this, not all ES cells were expressing Cre, leading to a heterogeneous cell population.

In conclusion, we efficiently recombined our FOG-1 donor vector in the pre-targeted R26^Hygro^ locus to generate R26^FOG-1^ ES cells and demonstrated that, upon Cre expression, both FOG-1 and hCD2t were expressed in these ES cells. We therefore used the R26^FOG-1^ ES cell clone to generate transgenic R26^FOG-1^ mice.

### Enforced expression of FOG-1 does not affect B-cell differentiation or function

**Figure 5:**
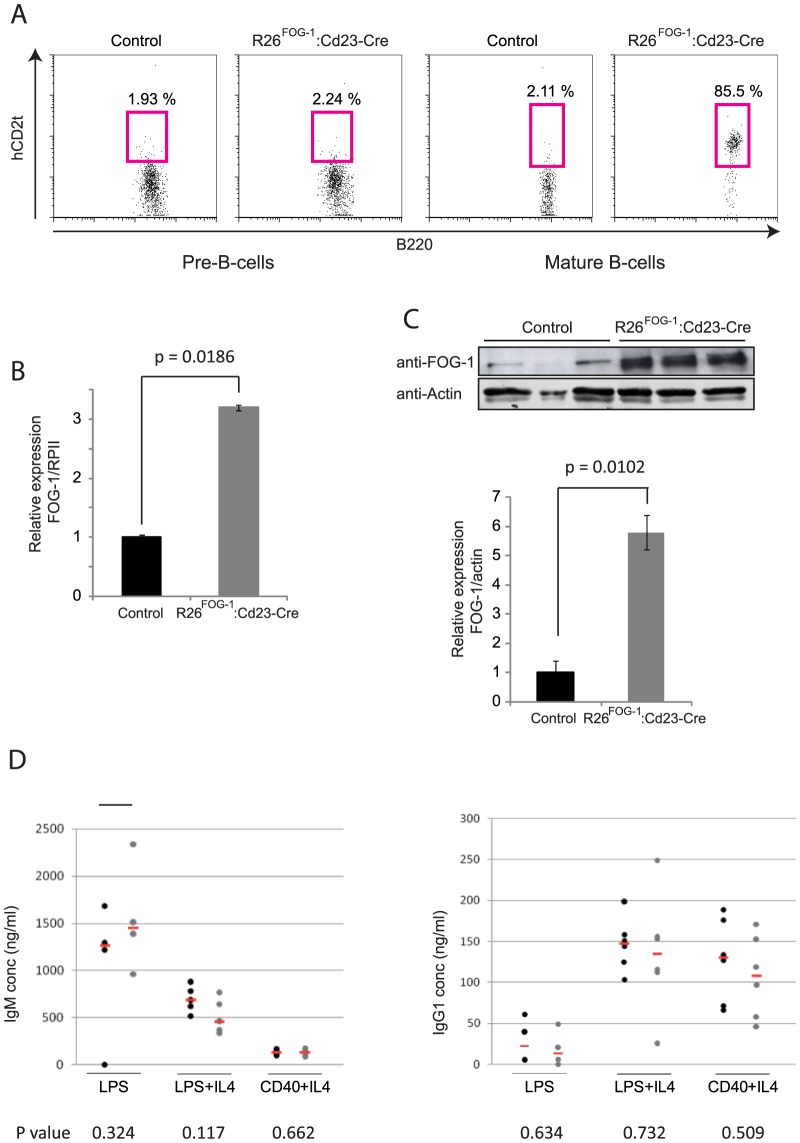
Overexpression of FOG-1 in mature B-cells. **A**. In R26^FOG-1^:Cd23-Cre mice, hCD2t expression is restricted to mature B-cells. Cell surface expression of hCD2t was analyzed by flow cytometry in Pre-B-cells (B220+, CD25+) and in mature B-cells (B220+, IgM^high^) of control (R26^FOG-1^) and R26^FOG-1^:Cd23-Cre mice. Representative results of at least 3 independent experiments are shown. **B**. FOG-1 mRNA is increased 3-fold in R26^FOG-1^:Cd23-Cre mature B-cells. RNA extracted from 3 control (R26^FOG-1^) and 3 R26^FOG-1^:Cd23-Cre mice was reverse transcribed and subjected to quantitative PCR to detect FOG-1 and RNA Polymerase II (RPII, for normalization) transcripts. Standard error of the mean is shown. **C**. FOG-1 protein is up-regulated ca. 6-fold in mature B-cells derived from R26^FOG-1^:Cd23-Cre mice. FOG-1 and actin proteins were detected by western blotting in mature B-cells derived from 3 control (R26^FOG-1^) and 3 R26^FOG-1^:Cd23-Cre mice (upper panel). The band intensities were quantified by LiCor Odyssey scanning and normalized to expression of actin (lower panel). Standard error of the mean is shown. **D**. FOG-1-overexpressing mature B-cells respond normally to *in vitro* stimulation. Splenic resting mature B-cells isolated from 3 to 6 control (R26^FOG-1^, black dots) or R26^FOG-1^:Cd23-Cre mice (grey dots) were activated *in vitro* by LPS, LPS+IL4 or anti-CD40+IL4 for 4 days. Titers of IgM (left panel) or IgG1 (right panel) in the culture supernatants were determined by ELISA; means are shown (red bar) as well as the corresponding p values at the bottom.

To investigate the potential effect of sustained level of FOG-1 in mature B-cells, we first crossed the R26^FOG-1^ mice with Cd23-Cre mice which express Cre specifically in mature B-cells [20]. Flow cytometry analysis of hCD2t expression in B-cells derived from R26^FOG-1^:Cd23-Cre mice showed that in the early developmental stages (Pre-B-cells), no hCD2t could be detected, as expected (Figure 5A, second panel). In contrast, more than 85% of the mature B-cells derived from these animals were found to express hCD2t (Figure 5A, fourth panel). Importantly, the expression of hCD2t was never detected in control animals which carry the R26^FOG-1^ allele but do not express Cre. Next, quantitative RT-PCR and western blot analysis were performed to estimate the level of FOG-1 overexpression in mature B-cells derived from R26^FOG-1^:Cd23-Cre animals and compared to that of R26^FOG-1^ animals. As shown in Figure 5B, FOG-1 mRNA was upregulated slightly more than three fold in the overexpressing cells, as compared to the control cells. At the protein level, the increase was even larger: careful quantification of the FOG-1 signal in relation to the expression of actin showed that the protein was upregulated about 6-fold (Figure 5C). Overall this analysis demonstrated that our system allows reliable overexpression of FOG-1 *in vivo*.

To investigate the potential biological effect of this elevated level of FOG-1 on plasma cell development, mature B-cells derived from R26^FOG-1^:Cd23-Cre mice were activated *in vitro* with different stimuli and the antibody titers in the culture supernatants were determined by ELISA. Irrespective of the stimulus used, no difference in the level of IgM or IgG1 was observed between overexpressing and control cultures, indicating that enforced expression of FOG-1 in mature B-cells did not impair or alter their ability to differentiate into antibody-secreting cells in vitro (Figure 5D). In addition, we also performed immunization experiments to test whether FOG-1 overexpression in mature B cells might have an impact on the immune response *in vivo*. For this, R26^FOG-1^:Cd23-Cre and control mice were immunized with DNP-KLH and the serum titers of antigen-specific immunoglobulins were measured by ELISA 8 and 15 days later. However, also in this case no significant difference was found (data not shown). Thus, although the expression of FOG-1 is normally downregulated during B cell development, enforcing expression of this factor at late B cell stages did not reveal any detrimental effect, contrary to our hypothesis. Finally, R26^FOG-1^ mice were also crossed with mb1-Cre mice, to induce FOG-1 overexpression from the earliest stages of B cell development onwards. However, this did not impact B cell development as examined by flow cytometric analysis (data not shown).

### Reduction of eosinophil numbers upon enforced expression of FOG-1

**Figure 6:**
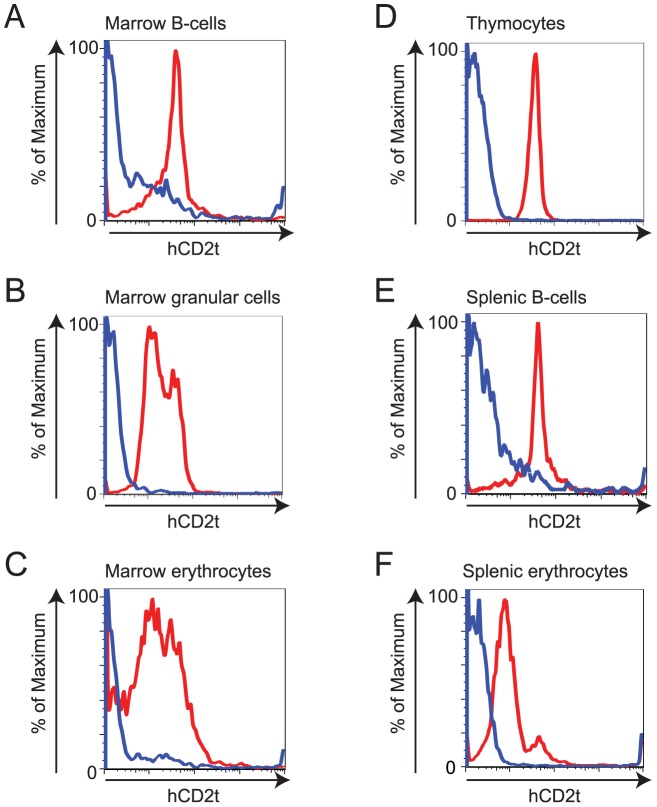
hCD2t is expressed in all hematopoietic cells of R26^FOG-1^:Vav-iCre mice. **A–F**. Flow cytometry analysis of hCD2t expression in R26^FOG-1^:Vav-iCre (red line) and control (R26^FOG-1^, blue line) mice. **A**. Bone marrow B-lymphocytes (B220+ cells). **B**. Bone marrow granular cells (based on Forward and Side Scatters). **C**. Bone marrow erythrocytes (TER119+ cells). **D**. Thymocytes (CD4+, CD8+ cells). **E**. Splenic B-lymphocytes (B220+ cells). **F**. Splenic erythrocytes (TER119+ cells). Data for one representative animal of each genotype are shown (n = 5).

To further analyze the consequences of an elevated level of FOG-1 in the hematopoietic system, we next crossed the R26^FOG-1^ mice with Vav-iCre mice [21] to overexpress FOG-1 in all hematopoietic lineages. Remarkably, all B-lymphocytes, myeloid cells, and erythrocytes derived from the bone marrow of R26^FOG-1^:Vav-iCre animals expressed hCD2t (Figure 6A, 6B and 6C). Similarly, all thymocytes and splenic B-lymphocytes as well as all splenic erythrocytes expressed hCD2t (Figure 6D, 6E and 6F), thus demonstrating the usefulness of our reporter system. Expression of transgene-derived FOG-1 mRNA was analyzed by quantitative RT-PCR and showed that FlagFOG-1 mRNA is produced at a similar level in bone marrow, spleen, and thymus of R26^FOG-1^:Vav-iCre mice (Figure S3). Altogether these data indicated that in R26^FOG-1^:Vav-iCre animals virtually all hematopoietic cells express the transgene at a roughly similar level.

**Figure 7:**
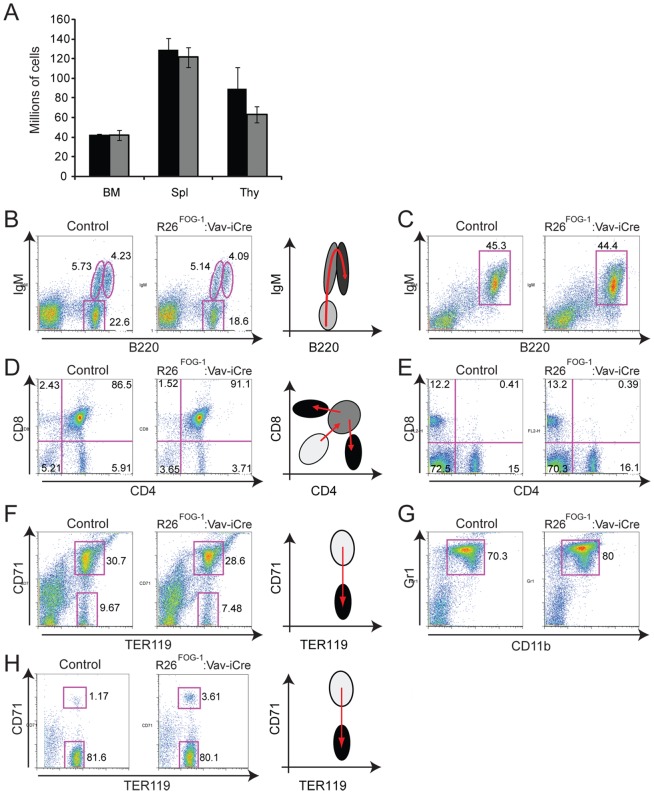
Normal B-cell, T-cell and granular cell populations in R26FOG-1:Vav-iCre mice. **A**. Cells of the bone marrow (BM), spleen (Spl) and thymus (Thy) of R26^FOG-1^ (black bars) and R26^FOG-1^:Vav-iCre (grey bars) mice were enumerated. Standard error of the mean is shown. **B**. Bone marrow cells were stained with anti-B220 and anti-IgM antibodies to analyze B-cell development. **C**. Splenocytes were stained with anti-B220 and anti-IgM antibodies to identify B-cells. **D**. Thymocytes were stained with anti-CD4 and anti-CD8 antibodies to analyze T-cell development. **E**. Splenocytes were stained with anti-CD4 and anti-CD8 antibodies to identify mature T-cells. **F**. Bone marrow cells were stained with anti-TER119 and anti-CD71 antibodies to analyze erythropoiesis. **G**. Bone marrow cells were stained with anti-Gr1 and anti-CD11b antibodies to identify Gr1+ CD11b+ myeloid cells. **H**. Splenocytes were stained with anti-TER119 and anti-CD71 antibodies to analyze splenic erythropoiesis. Cells were analyzed by flow cytometry in R26^FOG-1^ (control) and R26^FOG-1^:Vav-iCre animals; data for one representative animal are shown (n = 5 for each genotype). Percentages of the populations are shown next to the gates. A diagram representing the developmental pathway of the different lineages from pale (progenitors) to dark grey (differentiated cells) is shown next to the pseudo-dotplots B, D, F and H.

**Figure 8:**
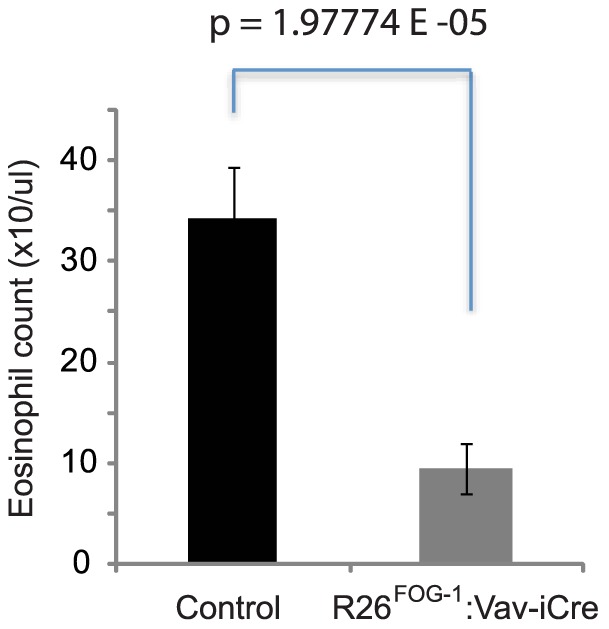
Altered eosinophil numbers in R26^FOG-1^:Vav-iCre mice. Reduction of circulating eosinophils. The numbers of eosinophils obtained in full blood count analysis of 8 control (R26^FOG-1^) and 8 R26^FOG-1^:Vav-iCre including those presented in Table 1 mice were averaged. Standard error of the mean is shown.

The total numbers of bone marrow cells, splenocytes, and thymocytes in overexpressing mice were comparable to the numbers obtained in control animals (Figure 7A). Using a panel of antibodies against lineage-specific surface markers we analyzed by flow cytometry the major hematopoietic cell populations. R26^FOG-1^:Vav-iCre mice showed normal B-cell development (Figure 7B and 7C), normal T-cell development (Figure 7D and 7E), as well as a normal myeloid population (Figure 7G). Developing erythrocytes can be subdivided into early and late erythroblasts based on the expression of the cell surface markers TER119 and CD71 [31]. Using this method, we found a largely normal erythroid development, although a slight but significant increase of the early erythroblast population (CD71+ TER119+) was seen in the spleen of some (≤50%) of the R26^FOG-1^:Vav-iCre animals (exemplified in Figure 7H). However, no difference was observed in the bone marrow (Figure 7F). More detailed analysis will be required to understand this phenotype. Statistical analysis of the flow cytometry data showed that, except for the increase in the early splenic erythroblast population (Figure 7H), expression of FOG1 did not have any significant impact on the cell populations examined (see Figure S4). Full blood count analysis of R26^FOG-1^:Vav-iCre females and males revealed no major difference in red blood cell count, hemoglobin content, hematocrit and platelet counts (Table 1). Similarly, no significant changes were observed in the total numbers of white blood cells, lymphocytes, monocytes, neutrophils and basophils in the blood of these mice (Table 1). In contrast, a consistent decrease in the total number of circulating eosinophils was observed for R26^FOG-1^:Vav-iCre females and males (Table 1). A highly reproducible and significant difference of more than 3-fold was found when comparing the average numbers of circulating eosinophils in control and R26^FOG-1^:Vav-iCre animals (Figure 8, p = 0.00758). To ascertain that the phenotypes observed were not due to Cre expression, which can have effects on its own in some cases [32], we also performed a flow cytometric and full blood count analysis of Vav-iCre mice in comparison with control animals lacking the Cre transgene (C57BL/6J). As can be seen in Figure S5, the flow cytometry profile of the different populations analyzed showed no difference between mice carrying the Vav-iCre transgene and control mice. Statistical analysis of the flow cytometry data demonstrated that there was indeed no significant difference between these mice (Figure S6). Furthermore, full blood count analysis also failed to show any statistically significant difference (Table S1). Finally, FOG1 mRNA expression in mature B cells was identical in C57BL/6J and Vav-iCre mice (Figure S7). Thus, the phenotypes described for the FOG1 overexpressing mice are not due to an artefact of cre expression, but are indeed FOG1-dependent.

**Table 1:** Full blood count of R26^FOG-1^:Vav-iCre.

	Control					R26FOG-1:Vav-iCre					
	Males		Females		Mean	Males		Females		Mean	P value
RBC (10∧4/uL)	1054	1032	979	1132	**1049**	1063	1136	1100	1067	**1091**	0.2981
HGB (g/L)	177	166	169	172	**171**	160	172	183	166	**170**	0.8965
HCT (10∧(−1)%)	534	505	508	516	**515**	492	525	532	507	**514**	0.8807
PLT (10∧3/uL)	1124	1352	914	1399	**1197**	1292	1241	1172	831	**1134**	0.6932
WBC (10/uL)	855	901	1595	1305	**1164**	653	573	1434	1314	**993**	0.5698
LYMPH (10/uL)	602	657	1205	970	**858**	485	412	1081	978	**739**	0.6083
MONO (10/uL)	150	130	255	207	**185**	75	106	230	226	**159**	0.6148
NEUT (10/uL)	79	83	87	92	**85**	82	49	107	103	**85**	1
BASO (10/uL)	0	0	1	0	**0**	0	0	0	2	**0.5**	0.6074
EO (10/uL)	24	31	47	36	**34**	*11*	*6*	*16*	*5*	***9***	*0.007589*

Blood samples from 4 control (R26^FOG-1^) and 4 R26^FOG-1^:Vav-iCre mice were examined with a mouse blood analyzer. Individual values are shown, as well as the corresponding averages (highlighted in bold) and the p values of the comparison between the 4 control and 4 FOG-1 expressing mice, determined by using Student's two-tailed t-test. Note the highly significant reduction of circulating eosinophils in FOG-1 overexpressing animals (highlighted in italic). RBC: red blood cells; HGB: hemoglobin; HCT: hematocrit; PLT: platelets; WBC: white blood cells; LYMPH: lymphocytes; MONO: monocytes; NEUT: neutrophils; BASO: basophils; EO: eosinophils.

## Discussion

We have shown here that FOG-1 expression is regulated during B cell development, being high in early stages (e.g. pre-B cells) and low or absent in late stages such as mature B cells and plasma cells. Based on these observations, we hypothesized that this downregulation is important for effective B cell development and that artificially maintaining FOG-1 expression at late stages might have an impact on normal B cell development or function. However, we did not observe any major phenotypes when FOG-1 was selectively overexpressed in mature B-cells. Flow cytometric analysis of the major B cell populations failed to reveal alterations in R26^FOG-1^:Cd23-Cre mice, demonstrating no obvious developmental impact. Furthermore, *in vivo* immune response to a T cell-dependent antigen or antibody secretion following *in vitro* stimulation were also not affected, thus suggesting that B cell function was not impaired. In addition, mice overexpressing FOG-1 from the pre-B cell stage onwards also did not show any remarkable phenotype. Nevertheless, it remains possible that FOG-1 affects a B cell subset that was not examined here and additional experiments will be required to determine this. Furthermore, since B cells do not express GATA factors (our unpublished data), other B cell ancillary factors must be postulated for a role for FOG-1 in these and potentially other cells (Versavel et al., in preparation).

In contrast, R26^FOG-1^:Vav-iCre mice with enforced expression of FOG-1 in the entire hematopoietic system showed two main phenotypes. First, consistent with the known role of FOG-1 in red blood cell development [4], [6], [33], [34], we observed a moderately altered erythropoiesis in the spleen of some (≤50%) R26^FOG-1^:Vav-iCre animals. The reason for the partial penetrance of this phenotype is unclear and additional work will be required. Second, we found a striking and highly significant reduction of the total number of circulating eosinophils. This is of great interest, since a previous study concluded that FOG-1 is a repressor of the eosinophil lineage in avian cells [12]. Our results, therefore, extend this finding to mammals. In the future, the R26^FOG-1^:Vav-iCre mice will be analyzed further to draw a more complete view of FOG-1 functions in hematopoiesis.

The Recombinase-Mediated Cassette Exchange technology relies on the exquisite selectivity of recombinases such as cre or flp and has been developed to facilitate the generation of transgenic ES cell lines [29], [35] [35]–[41] [15], [16]. To allow inducible or lineage-specific expression, the RMCE technology has been combined with tetracycline-inducible systems [42]–[44], or with the Cre recombinase activity [45], [46]. Recent studies made use of a pre-targeted locus to generate shRNA-expressing mice [47], [48] and Hitz *et al.* combined the C31 integrase-mediated recombination at a pre-targeted locus with Cre-dependent expression to establish shRNA-expressing mice [49]. Other systems that allow RMCE into pre-modified ES cells [50], [51] or in human ES cells pre-targeted at the *HPRT* locus [52] have also been developed.

To alter the expression pattern of FOG-1 *in vivo*, we developed a novel and rapid transgenic system that is also based on the RMCE technology and that allows rapid insertion of expression cassettes into the Rosa 26 locus. In this system, expression of the transgene from the endogenous *Rosa26* promoter is dependent on Cre recombinase-mediated excision of a STOP sequence, allowing cell- or temporal-specific control of expression. A hCD2t cDNA is also included as a reporter to track transgene-expressing cells *in vitro* and *in vivo*.

Our strategy is broadly similar to the one described by Hitz and colleagues, where C31 integrase-mediated recombination is used instead of FLP-mediated recombination to insert a transgene at a modified *Rosa26* locus and where the loxP/Cre system is used for conditional expression of shRNAs [49]; the RMCE efficiency of the two systems is also comparable. A particularly useful feature of our system is the concomitant expression of a “marker” gene, hCD2t, together with our transgene (i.e. FOG-1); this allows to monitor Cre-recombined cells *ex vivo* or *in vivo*. This is especially useful in situations where Cre expression, and therefore transgene expression, is only partial or mosaic and leads to a mixture of recombined and non-recombined cells; having a marker gene such as hCD2t then allows to selectively examine –and potentially isolate- the transgene-expressing cells. Expression of hCD2t can conveniently be detected by flow cytometry and is therefore particularly well suited for analyses in the hematopoietic system. Furthermore, since the antibodies recognizing hCD2t are species-specific, expression of the endogenous mouse protein (e.g. by T cells) does not interfere. Finally, hCD2t can also be detected by immunohistochemistry, further extending the range of cells that can be selectively examined.

Using this system we generated mice with moderate overexpression of FOG-1 either in mature B cells (R26^FOG-1^:Cd23-Cre mice), throughout B cell development (R26^FOG-1^:mb1-Cre), or in all hematopoietic lineages (R26^FOG-1^:Vav-iCre mice). Remarkably, flow cytometry analysis of bone marrows and spleens derived from R26^FOG-1^:Vav-iCre mice revealed that all hematopoietic cells expressed hCD2t. In contrast and as expected, in R26^FOG-1^:Cd23-Cre mice, where Cre starts being expressed just before the mature B-cell stage, hCD2t was only detected in mature B-cells. Importantly, cells derived from control R26^FOG-1^ mice lacking Cre expression did not express any hCD2t. These results demonstrate that conditional expression of a transgene using our system is tightly regulated and underscore the utility of having a marker gene.

We found that the *Rosa26* promoter drives moderate FOG-1 expression in the hematopoietic system. Interestingly, this expression level was sufficient to marginally alter splenic erythropoiesis and to significantly reduce the number of circulating eosinophils in R26^FOG-1^:Vav-iCre mice, demonstrating its physiological relevance. Such moderate expression level is an advantage to unravel the roles of proteins in physiologic and pathologic situations, as it avoids aberrant phenotypes that may be partly caused by too strong overexpression. Since targeted *Rosa26* homozygous mice are viable and appear normal, the expression level of the transgene could be doubled by generating R26^FOG-1^/R26^FOG-1^ homozygous mice [18] or by inserting additional regulatory elements in the expression cassette. Following this idea, Tchorz *et al.* generated a modified RMCE-compatible *Rosa26* locus for the expression of transgenes and characterized several promoters with different strengths [17].

In conclusion, despite our finding that the expression of FOG-1 is tightly regulated throughout B-cell differentiation and is dependent on the B-cell specific coactivator OBF1, we could not demonstrate a role for FOG-1 in B-cell differentiation or function. However, we could confirm the finding that FOG-1 is a negative regulator of eosinophil development and extend it to mammals. Further work will be required to better understand this important function of FOG-1 in the mouse.

## Supporting Information

Figure S1 Pre-targeting of ES cells with the pR26-SA-FRT-Hygro^R^ vector. **A**. Schematic representation of the wild-type and pre-targeted R26^Hygro^ alleles. The splice acceptor (yellow dot) and the hygromycin B resistance cassette (Hygro, orange) flanked by FRT sites were inserted using the homology arms (thick grey) between exons 1 and 2 (blue). The PCR primers (0F, 0R; arrows) as well as the restriction sites and probes (green bars) used for Southern blotting are shown. For clarity, only the relevant PstI sites are shown. **B**. PCR screening of the putative R26^Hygro^ ES cell clones. Clones 1–4 are positive, clone 5 shows an aberrant product. **C–E**. Correct insertion of the transgene confirmed by Southern blotting. Clone 5 was included as a negative control. To test the 5′ insertion, BamHI-digested ES cell genomic DNA was hybridized with the radioactively labeled 5′ probe to detect the wild-type (WT, 5.8 kb) and the targeted (Targ, 4.9 kb) bands (**C**). To test the 3′ insertion, PstI-digested DNA was hybridized with the radioactively labeled 3′ probe to detect the 6.5 kb WT and the 7.5 kb targeted bands (**D**). To verify single-copy insertion, PvuII-digested DNA was hybridized with a radioactively labeled internal probe to detect the 8 kb targeted band (arrow). Note that clone 1 shows an aberrant extra band, indicating multiple insertions in this clone (**E**).
(EPS)

Figure S2 Efficiency of RMCE at the pre-targeted R26^Hygro^ allele. **A**. Schematic representation of the different alleles, from top to bottom: wild-type R26 locus, R26^Hygro^, R26^Control^ and R26^FOG-1^. The different primer pairs used for PCR analysis of the ES clones are depicted by arrows. **B**. PCR analysis of Neo^R^/Hygro^S^ ES cell clones for testing RMCE recombination at the 5′ (FRT3) and at the 3′ (FRTwt) sites. Lanes 1–12: control clones, cells derived from RMCE with the control donor vector; lanes 1′–12′: FOG-1 clones, cells derived from RMCE with the FOG-1 donor vector; + Ctl, positive control. From top to bottom: PCR screening with primer pairs 1F/1R and 3F/3R at the 5′ end junction of the recombined cassette. PCR screening with primer pairs 2F/2R and 4F/4R at the 3′ end junction of the recombined cassette. Appropriate positive controls were chosen for each PCR set up. Note that on the presented gel control clone 9 shows a faint band with primer pair 3F/3R. Upon reanalysis of the DNA it was however found to be positive only with primers 1F/1R, as would be expected from a correctly recombined clone. **C**. PCR analysis of Neo^R^/Hygro^S^ ES cell clones for presence of the Neomycin resistance gene; -Ctl, negative control. **D**. PCR analysis of Neo^R^/Hygro^S^ ES cell clones for presence of the human CD2t gene. Lanes labeling as in (B) above. As shown, all clones analyzed are positive for both the Neomycin and the hCD2t gene.
(EPS)

Figure S3 Expression of transgene-derived FOG-1 in R26FOG-1:Vav-iCre animals. Total RNA from bone marrow (BM), spleen (Spl), and thymus (Thy) of 3 R26^FOG-1^:Vav-iCre animals was extracted, reverse transcribed and subjected to quantitative PCR to specifically detect transgene-derived FlagFOG-1 mRNA. Values are relative to RNA Polymerase II (RPII) expression. Standard error of the mean is shown. FlagFOG-1/RPII relative expression in bone marrow was arbitrarily set to 1.
(EPS)

Figure S4 Statistical analysis of the flow cytometry data.The flow cytometric data of R26^FOG-1^ (blue bars) and R26^FOG-1^:Vav-iCre (red bars) animals (including the mice presented in Figure 7) were used for statistical analysis applying Student's two-tailed t-test. **A**. Bone marrow B-cells. **B**. Bone marrow myeloid cells. **C**. Bone marrow erythroid cells. **D**. Splenic B-cells. **E**. Splenic mature T-cells. **F**. Splenic erythropoiesis. **G**. Thymocytes.
(EPS)

Figure S5 Normal B-cell and granular cell populations in Vav-iCre mice. **A**. Cells of the bone marrow (BM), spleen (Spl) and thymus (Thy) of control (C57BL/6J, blue bars) and Vav-iCre (red bars) mice were enumerated. Standard error of the mean is shown. **B**. Bone marrow cells were stained with anti-B220 and anti-IgM antibodies to analyze B-cell development. **C**. Splenocytes were stained with anti-B220 and anti-IgM antibodies to identify B-cells. **D**. Bone marrow cells were stained with anti-TER119 and anti-CD71 antibodies to analyze erythropoiesis. **E**. Bone marrow cells were stained with anti-Gr1 and anti-CD11b antibodies to identify Gr1+ CD11b+ myeloid cells. **F**. Splenocytes were stained with anti-TER119 and anti-CD71 antibodies to analyze splenic erythropoiesis. Cells were analyzed by flow cytometry; data for one representative animal are shown (n = 4 for each genotype). Percentages of the populations are shown next to the gates. The statistical analysis (two-tailed Student's t-test) of the data is presented.
(EPS)

Figure S6 Statistical analysis of the flow cytometry data in Vav-iCre mice. The flow cytometric data presented in Figure S5 of control (C57BL/6, blue bars) and Vav-iCre animals (red bars) were used for statistical analysis applying Student's two-tailed t-test. **A**. Bone marrow B-cells. **B**. Bone marrow erythroid cells. **C**. Bone marrow myeloid cells. **D**. Splenic B-cells. **E**. Splenic erythropoiesis.
(EPS)

Figure S7 Unchanged FOG1 expression in Vav-iCre mice. RNA from mature B cells from C57BL/6J (WT) or Vav-iCre mice (n = 4 per genotype) was used to measure FOG1 mRNA expression by RT-qPCR. Statistical analysis was done using Student's two-tailed t-test.
(EPS)

Table S1 Blood samples from 4 control (C57BL/6J) and 4 Vav-iCre mice were examined with a mouse blood analyzer. Individual values are shown, as well as the corresponding averages (in red) and the p values of the comparison between the 4 control and 4 Cre expressing mice. Note the absence of significant variation. RBC: red blood cells; HGB: hemoglobin; HCT: hematocrit; PLT: platelets; WBC: white blood cells; LYMPH: lymphocytes; MONO: monocytes; NEUT: neutrophils; BASO: basophils; EO: eosinophils.
(DOCX)
