# Supplementary material for: Hematopoietic Overexpression of FOG1 Does Not Affect B-Cells but Reduces the Number of Circulating Eosinophils
Source: PLoS One. 2014 Apr 18;9(4):e92836. doi: 10.1371/journal.pone.0092836 (PMC3991581; doi:10.1371/journal.pone.0092836)
Supplement: Table S1 — Blood samples from 4 control (C57BL/6J) and 4 Vav-iCre mice were examined with a mouse blood analyzer. Individual values are shown, as well as the corresponding averages (in red) and the p values of the comparison between the 4 control and 4 Cre expressing mice. Note the absence of significant variation. RBC: red blood cells; HGB: hemoglobin; HCT: hematocrit; PLT: platelets; WBC: white blood cells; LYMPH: lymphocytes; MONO: monocytes; NEUT: neutrophils; BASO: basophils; EO: eosinophils. (DOCX) [file pone.0092836.s008.docx]

**Supporting Table 1 Du Roure et al.**

|  | Control | | | |  | Vav-iCre | | | |  |  |
| --- | --- | --- | --- | --- | --- | --- | --- | --- | --- | --- | --- |
|  | **Males** | | **Females** | | **Mean** | **Males** | | **Females** | | **Mean** | **P value** |
| RBC (10^4/uL) | 882 | 817 | 1034 | 953 | **922** | 828 | 818 | 950 | 910 | **877** | 0.457 |
| HGB (g/L) | 158 | 135 | 169 | 158 | **155** | 141 | 131 | 160 | 148 | **145** | 0.328 |
| HCT  (10^(-1)%) | 412 | 390 | 493 | 472 | **442** | 404 | 390 | 454 | 432 | **420** | 0.470 |
| PLT (10^3/uL) | 142 | 341 | 1013 | 483 | **495** | 233 | 539 | 1058 | 960 | **698** | 0.477 |
| WBC (10/uL) | 1145 | 1096 | 810 | 1150 | **1050** | 1510 | 1458 | 526 | 876 | **1093** | 0.872 |
| LYMPH (10/uL) | 841 | 880 | 567 | 865 | **788** | 1217 | 1072 | 376 | 682 | **837** | 0.820 |
| MONO (10/uL) | 140 | 113 | 150 | 164 | **142** | 133 | 93 | 76 | 88 | **98** | 0.036 |
| NEUT (10/uL) | 138 | 60 | 82 | 87 | **92** | 118 | 267 | 62 | 80 | **132** | 0.449 |
| BASO (10/uL) | 1 | 2 | 1 | 1 | **1** | 1 | 1 | 1 | 0 | **1** | 0.207 |
| EO (10/uL) | 25 | 41 | 10 | 33 | **27** | 41 | 25 | 11 | 26 | **26** | 0.873 |
